# A dataset of logistics sites in England and Wales: Location, size, type and loading bays

**DOI:** 10.1016/j.dib.2024.110399

**Published:** 2024-04-16

**Authors:** Christopher de Saxe, Daniel Ainalis, David Cebon

**Affiliations:** a Department of Engineering, University of Cambridge, Trumpington Street, Cambridge CB2 1PZ, United Kingdom; b University of the Witwatersrand, School of Mechanical, Industrial and Aeronautical Engineering, Private Bag 3, Wits, Johannesburg 2050, South Africa

**Keywords:** Heavy goods vehicles, Warehouses, Retail, Factories, Freight, Transport, Electrification

## Abstract

Data on the location and size of logistics sites is essential for the accurate system-level modelling of transport and logistics operations. This is becoming increasingly important to support governments and industry transition to a net zero future which will feature new operating models and vehicle technologies, particularly for electric vehicle operations. In this work we present a dataset of logistics sites across England and Wales categorised into warehouses, retail sites, and factories. There are 47,683 rows of data in total, comprising 27,691 warehouse sites, 6,441 retail sites, and 13,551 factory sites. Each row contains the site's category, location (latitude and longitude), size (in square meters), and modelled number of heavy goods vehicle loading bays. Raw data on non-domestic properties in England and Wales were sourced from the UK's Valuation Agency Office database. Addresses were geocoded to determine the coordinates of each site, floor area was determined for each site via a web crawler script, and the type of site was derived using a keyword-based categorisation process. The size of the site gives an indication of the expected transport activity (i.e. volume of goods handled) and is a useful proxy to estimate the number of loading bays which, in turn, is a useful proxy for the number of electric heavy goods vehicle charging points the site may have to accommodate to support electric vehicle operations. Models relating the floor area to the number of loading bays were developed using satellite imagery of a sample of data from each category. Uncertainty in the geolocation, category and floor area data is deemed to be very low<1%), while the models to predict loading bay data are based on a sample of the overall dataset and subject to higher uncertainty (<20 %). Larger sample datasets and alternative models may be explored in future work to suit other applications.

Specifications Table

**Table:** 

Subject	Business, Management and decision sciences
Specific subject area	Transportation management
Data format	processed, analysed
Type of data	Table (.csv)
Data collection	Raw data on non-domestic properties in England and Wales were sourced from the UK's Valuation Agency Office database. Addresses were geocoded to determine the coordinates of each site, floor area was determined via a web crawler script, the type of site was derived using a keyword-based categorisation process, and the number of loading bays was modelled using satellite imagery of a sample of the data.
Data source location	The processed dataset is stored in Apollo, the institutional repository of the University of Cambridge [1]. Raw data were sourced from the UK Valuation Agency Office rating list [2] (‘2023 non domestic rating list entries’ – exportable as a .csv file) and the related property search website [3].
Data accessibility	Repository name: Apollo Data identification number: 10.17863/CAM.102176 Direct URL to data: https://doi.org/10.17863/CAM.102176

## 1 Value of the Data

• The dataset supports the transition to electrified road freight transport, wherein logistics facilities (and the loading bays in particular) will become important opportunity charging sites for electric Heavy Goods Vehicles (‘eHGVs’). The dataset will help (i) operators plan and optimise future eHGV operations to inform vehicle and depot charging investment decisions, (ii) logistics site owners forecast the demand for charging at their sites to plan infrastructure investment, (iii) government and electricity network operators forecast increased electricity grid loading and grid connection requests for new eHGV chargers, and (iv) government and academics forecast the total cost and emissions impact of national eHGV charging infrastructure.
• Related to this, the dataset is valuable for studies involving dynamic charging infrastructure or ‘electric road systems’ (ERS). Such a system will likely co-exist with static charging infrastructure (opportunity and overnight) and a trade-off will exist. The dataset will enable various scenarios of both dynamic and static charging infrastructure to be studied and the cost and planning implications to be assessed and optimised.
• Third-party charging infrastructure providers can use the data to predict where public charging sites may be needed, based on a forecast availability of other charging sites.
• The dataset can be used in system-level modelling of UK logistics operations, especially in agent-based modelling studies. The location data provide the origin and destination of the majority of logistics journeys, while the category and number of loading bays gives an indication of the type and volume of freight handled and hence the number of HGV visits at each site.
• Opportunities for multimodal logistics operations can be determined by overlaying the dataset with data on rail-sidings, ports, and large depots.

## 2 Data Description

In this work we present a dataset of UK logistics sites which includes the location, size (in square meters) and number of loading bays at each site. The size provides an indication of the expected activity of the site (i.e. volume of goods handled), but is also a useful proxy for the number of loading bays. The number of loading bays is, in turn, a useful proxy for other variables such as the volumes of freight processed and number of charging sites for future electric HGVs. The latter application is of particular interest to further studies on both static and dynamic charging of eHGVs in the UK [4], [5], [6], [7] and related studies in Europe [8], [9], [10], [11].

The dataset [1] represents logistics sites across England and Wales. The term ‘logistics site’ is used here to broadly represent any site where HGVs will be required to stop to load or unload goods or materials, especially at a dedicated loading bay.

The data are split into three high-level categories of logistics sites, namely: warehouse, retail, and factory. These categories were chosen as they capture all primary logistics site types while sufficiently delineating between distinct loading bay characteristics of each. Warehouses include distribution and consolidation centres, and so will have both HGV loading and unloading activities. Depending on the size of the site, these activities may take place at separate dedicated loading bays or shared loading bays. Likewise, factory sites are both loading and unloading locations (for raw materials/components and finished goods, respectively), though typically have far fewer loading bays compared to warehouse sites. Again, the size will determine whether there are separate loading and unloading bays or not. Finally, most retail sites will only have one loading bay for receiving finished goods.

**Fig. 1:**
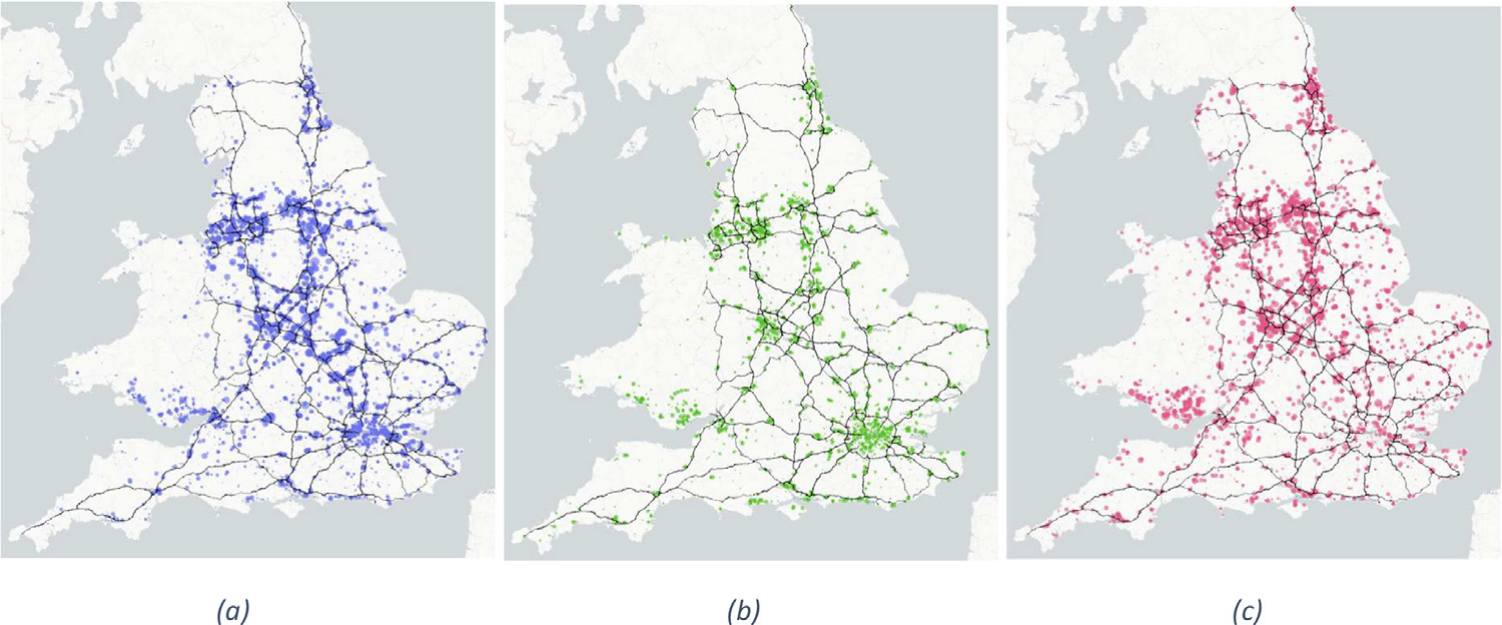
The warehouse (a), retail (b), and factory (c) data points visualised on a map of England and Wales. The data are shown alongside England's strategic road network for reference. Each bubble is scaled relative to the total floor area in square meters.

There are 47,683 rows of data in total, comprising 27,691 warehouse sites, 6,441 retail sites, and 13,551 factory sites. The dataset comprises five columns including: the category of the facility; the latitude and longitude coordinates of the entry; floor area in square metres; and the estimated number of loading bays according to the models in Section 3. The variables are summarised in Table 1. The dataset is visualised in Fig. 1, showing each of the categories in isolation, superimposed on the strategic road network for reference.

**Table 1:** Description of variables in the dataset.

Name	Description	Data type	Units	Source
category	Keyword defining type of site. Values are ‘warehouse’, ‘factory’, ‘retail’	Discrete	–	Processed from VOA data [2] using a keyword search
latitude	Latitude coordinate of the site	Float	Degrees (°)	Geocoded from VOA addresses [2]
longitude	Longitude coordinate of the site	Float	Degrees (°)	Geocoded from VOA addresses [2]
sqm	The floor area of the site as used for valuation purposes	Float	Square meters (m^2^)	Obtained from the VOA property search webpage [3] using a webcrawler
bays	The modelled number of loading bays at the site based on a sample of sites with a known number of loading bays.	Integer	–	Modelled from satellite imagery (see section 3.3)

## 3 Experimental Design, Materials and Methods

This section describes the steps taken to collect and process the data, along with the modelling procedure to estimate the number of loading bays. A discussion about the limitations of the dataset is presented, highlighting areas of caution and avenues of further development and improvement.

### 3.1 Raw data collection

Raw data were collected from the UK government's property rates database [2]. This includes valuation data for business premises across England and Wales as determined by the Valuation Office Agency (VOA). The ‘2023 non domestic rating list entries’ dataset was used. Alongside the valuation itself, the data contain street addresses, descriptions according to set category names, and a special category code. The entire database was exported to a CSV file which included 2143,080 rows and 29 columns/fields of data, totalling over 500 MB.

Additional data including a breakdown of floor area used in the rating calculation is available in the source database via the online search interface on a per-entry basis [3]. This includes a table of individual floor areas, their valuation rate (£/m^2^), the total valuation per floor area, and the sum total valuation of all floor areas. Most entries contain floor area information, especially the larger sites, but there were some entries without the area information.

### 3.2 Data processing

The raw CSV file contains valuable address and description data, but these are not usable in their current form for further modelling and visualisation. Furthermore, the exportable dataset excludes the highly relevant floor area data which is only available via the manual online search interface.

Hence the raw data required the following processing steps to be converted into a useable state in this project:

1. Geocode street-level addresses from the VOA dataset [2] into location coordinates (latitude and longitude).
2. Obtain and integrate floor area data from the VOA property search website [3].
3. Process the description field to remove irrelevant entries and categorise the remaining entries into one of: warehouse, retail, and factory sites.
4. Remove all entries below a threshold floor area size.

Each of these steps will be discussed in turn in the following subsections.

### 3.3 Geocoding

The raw dataset contains address information including street number, street, town, village, post code, and county. In some cases, the company name is included as well. This information must be converted into location coordinates (longitude and latitude) in order for it to be visualised and used in quantitative geographical processing (i.e. measuring proximity to the road network, electricity grid, or other relevant sites). A Python script was prepared for this purpose which reads the address information for each entry and returns the latitude and longitude coordinates. The script makes use of the ‘geopy’ Python client [12].

Initially, both the ‘Nominatim’ [13] and ‘GoogleV3’ [14] geocoding services were used with the ‘address’ field as an input. The results were analysed to verify the reliability of the data by checking for failed geocoding instances (with no value returned) or co-ordinates which exceeded the known extreme North, South, East and West boundaries of England and Wales (taken to be latitudes of 49.882 to 55.811° and longitudes of −6.449 to 1.763°). An error rate of 5.5% was observed, which was reasonable given the quality of the address data, but insufficient for the current application.

The geocoding was then repeated using only the ‘postcode’ field and the GoogleV3 geocoding service, returning a 100% success rate against the above metrics. While the postcode centroid is less precise than the exact address, the substantial increase in reliability with a small loss of precision was deemed preferable for this application. There are approximately 1.79 million postcodes in the UK covering around 243,610 km^2^ of land area. This gives an average post code size of 0.136 km^2^ per postcode. Noting that the largest UK post code areas are outside of England and Wales, the average area for the current dataset will be smaller and suitable for the current application.

### 3.4 Floor area data

While the floor area data is available via the online search interface [3], it is not included in the downloadable CSV file [2]. Given the sheer size of the dataset, obtaining these data manually from the website would not be feasible. Fortunately, every entry in the CSV file contains a unique identifier which facilitates the automation of this process as follows.

The web page for each entry is defined by the URL base https://www.tax.service.gov.uk/business-rates-find/valuations/start/ followed by the identifier number. For example, the identifier for the ‘University Of Cambridge Dept Of Engineering & School Of Architecture, Trumpington Street, Cambridge, CB2 1QA’ [sic] is 11,242,919,000, and so the URL for this entry is simply:

https://www.tax.service.gov.uk/business-rates-find/valuations/start/11242919000

The information of interest is the total floor area. In the underlying HTML code of each entry webpage, this value was found to be consistently contained in a table cell with the ID ‘floor-lines-total-area’. A python ‘web crawler’ script was prepared to automate the process of compiling the necessary URL and returning the floor area value for each entry, using the ‘requests’ [15] and ‘BeautifulSoup’ [16] libraries. This value constitutes the third field in the final dataset.

As these data are directly government sourced, their accuracy will be subject to government valuation processes. It should be noted however that these *floor area* data are not necessarily the same as the *footprint* of a site, the latter being better potentially better correlated with loading bays. While the two figures will often be identical or close, larger deviations are expected for multistorey sites. While some multistorey sites will exist in the dataset (and some of these are highlighted later), it is expected that these will be in the minority and that the valuation floor area represents a suitably robust indicator of loading bays.

### 3.5 Categorisation

The raw dataset contains a useful set of fields which describe the type of business premises according to a ‘primary and secondary description code’, ‘primary description text’, and ‘special category code’. The most useful of these was found to be in the ‘primary description text’ field.

A list of ‘primary description default descriptions’ and ‘overtyped descriptions’ are provided in the ‘technical guidance’ for the VOA Rating List [2], totalling around 1000 possibilities. In reality, the text within the ‘primary description text’ contains several hundred more variations and combinations of these, some more specific to certain industries, and often with subtle wording and spelling variations and spelling errors. For example, in the context of warehouse sites, the most common description text found in the dataset is ‘WAREHOUSE AND PREMISES’. However, many other variations of this exist which are of interest for our purposes, including:

• WAREHOUSE & PREMISES
• WAREHOUSE, OFFICE AND PREMISES
• WAREHOUSE,OFFICES,LAND & PREMISES
• COLD STORE & PREMISES
• LABORATORY WAREHOUSE AND PREMISES
• RAILHEAD AND DISTRIBUTION CENTRE
• DISTRIBUTION CENTRE AND PREMISES

Other variations exist which are not relevant to our needs, which need to be excluded, such as:

• DAY NURSERY,WAREHOUSE AND PREMISES
• OFFICES WAREHOUSE AND PREMISES
• WORKSHOP WAREHOUSE & PREMISES
• NUSERY, WAREHOUSE, RESTAURANT AND PREMISES (PART EXEMPT)

Given these complications and variations, the process of finding entries relevant for our purposes could not be reliably automated, and so a combined keyword search and manual outlier rejection and categorisation process was followed:

1. **Keyword search**: All entries relevant to one of our three target categories (warehouse, retail, factory) were extracted from the raw dataset using MATLAB. Keywords used included ‘warehouse’, ‘cold store’, ‘distribution’, ‘production’, ‘factories’, ‘industrial’, ‘depot’, ‘storage’, ‘superstore’, ‘supermarket’, and ‘manufacturing’.
2. **Outlier rejection**: Following this, non-relevant entries which were picked up during the keyword search were manually removed.
3. **Categorisation**: Finally, the remaining entries were manually categorised into one of the three final categories.

The initial set of keywords was created by creating a list of all unique terms in the ‘primary description text’ field, and a review of all dominant words and their spelling variations. For thoroughness, keywords which might capture non-relevant terms were also included, as the non-relevant terms would be manually removed in the next step. For example, the keyword “depot” was included to capture terms such as “distribution depot”, but would also capture the less-relevant “bus depot” which would then be manually filtered out. Through this process the keyword search process was deemed sufficiently robust for application here. In any case, the dataset was dominated by only a handful of descriptors with obvious keywords. Any uncertainty in some of the more obscure keywords would impact less than 0.01% of the data. (See Appendix A: Supplementary material).

The three categories (warehouse, factory, retail) were chosen to balance simplicity with the need to sufficiently delineate between loading bay characteristics. For example, a warehouse, distribution centre, or cold store were observed to exhibit a similar number of loading bays per square meter. Likewise, “factory” sites were observed to possess a largely similar need for loading bays for raw materials delivery and finished goods distribution, irrespective of it being an industrial bakery, auto parts manufacturer, or consumer goods manufacturer. These choices were supported by an analysis of the most frequent terms found in the primary description text field. Further granulation (such as “cold store” and “distribution centre”) was not found to have an observable impact on the loading bay trends and was deemed unnecessary for the current purpose. Other common categories in the dataset like “Offices and premises” were excluded as being unlikely to have a need for a dedicated HGV loading bay. Other categorisations may be carried out for other applications as needed.

From observation, it was found that the leading terms in the ‘primary description text’ were the most reliable indication of site category. For example “warehouse brewery office and premises” is most likely to be a warehouse site serving a brewery with a small attached office site, whereas, “brewery warehouse, office and premises” was most likely to be a brewery (hence “factory”) site with a small warehouse facility and/or office attached. Based on this it was also decided that each site would be best categorised by one category based on its leading term, rather than grouping sites into multiple categories based on all terms in the descriptor.

What may also be evident from these examples is the inconsistent inclusion/exclusion of commas in the descriptors coupled with spelling variations which can give rise to ambiguity. While it was not possible to fully predict the intended meaning in the dataset, but the leading term approach is the most robust method given this uncertainty. Furthermore, in each of the three categories, 99% of the entries are described by only ten descriptors, with over 90% being defined by just one or two descriptors:

• “warehouse and premises”- 92.62% of the warehouses dataset
• “retail warehouse and premises” and “superstore and premises” – 91.45% of the retail dataset
• “factory and premises” - 96.47% of the factory dataset

The final list of filtered descriptors by category is given in Appendix A: Supplementary material which includes the frequency and relative frequency of each descriptor text.

### 3.6 Removing smaller sites

**Fig. 2:**
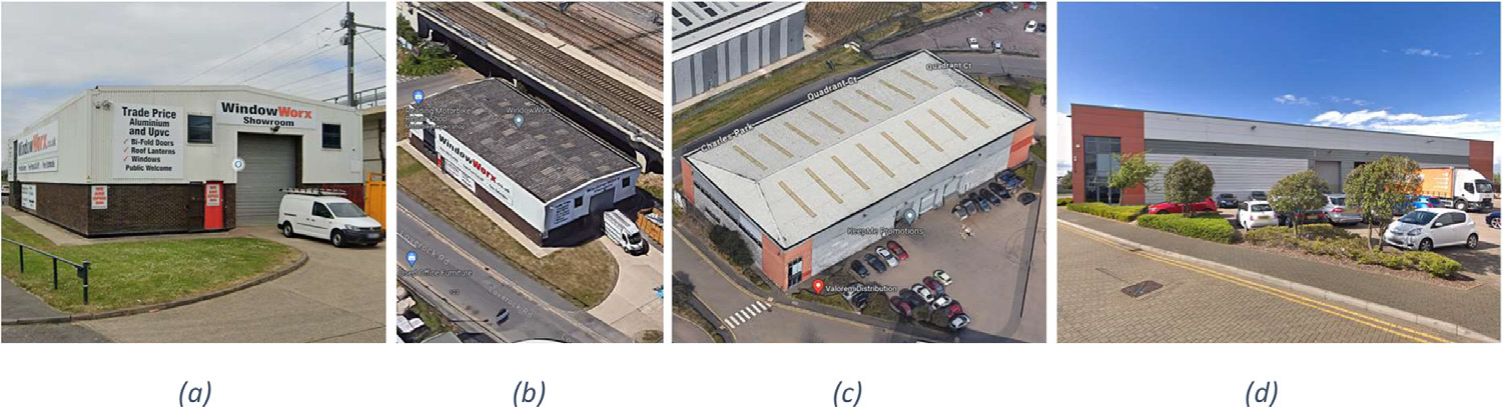
Google StreetView and satellite image examples of sites below and above the 1000 m^2^ threshold: (a, b) 60 Loverock Road, Reading (478 m^2^), with limited driveway area and evidence of being serviced by light vans. (c, d) 1 Quadrant Court, Crossways Business Park, Dartford (1978 m^2^) with several large loading doors and evidence of HGV use.

Finally, smaller sites were not considered relevant to HGV logistics operations, as they would typically be serviced only by light and medium goods vehicles or vans, and typically without the use of a dedicated loading bay. As such, the dataset was limited to sites with a floor area of 1000 m^2^ or more. The 1000 m^2^ threshold was determined based on the inspection of sample data points on Google Maps and expert opinion. For sites below 1000 m^2^, sites were more likely to either not have a loading bay or to be serviced only by a van or light commercial vehicle. For sites above 1000 m^2^, they were more likely to have a loading bay suited to an HGV. Two representative examples are provided in Fig. 2 to demonstrate.

This had the additional benefit of reducing the dataset to a more manageable size (from 171,084 to 47,683 across all categories) for visualisation and further processing. Should similar data be needed for smaller logistics sites, the process presented in this paper can easily be re-applied using an alternative threshold.

### 3.7 Loading bay estimation

While data on the size of a logistics site is useful for many applications, the number of loading bays is particularly helpful for understanding and modelling HGV activities within the logistics system. Chief amongst the use cases at present is the design and roll-out of eHGV charging facilities at these sites, which would likely focus on loading bays so that vehicles could charge during the downtime of loading/unloading.

It was hypothesized that the number of loading bays at a logistics site would be approximately correlated with its size in square meters, but that this correlation would be different for each of to the three major categories of sites: warehouses, factories and retail sites. To determine an appropriate correlation, the following process was carried out per category:

1. A sample of sites across the spectrum of floor areas were selected from the dataset.
2. Each site was located on Google Maps, and its number of loading bays was counted from satellite imagery.
3. The approximate footprint area of the site was measured for comparison with VOA data.
4. Outliers were identified in the sample data based on:
  a. Being a known multistorey site.
  b. A large discrepancy between measured and VOA footprint (likely a multistorey site).
  c. Visually out of trend with the other data (due to an atypical number of loading bays).

The stratification used was not constant across the VOA area spectrum. Rather, a finer spacing between samples was used for data with smaller areas to account for the higher numbers of data points at the lower end of the area. A reasonably linear variation in spacing as a function of area was used within the practical constraints of certain sites not being clearly visible in satellite imagery, for example.

#### 3.7.1 Data collection

**Fig. 3:**
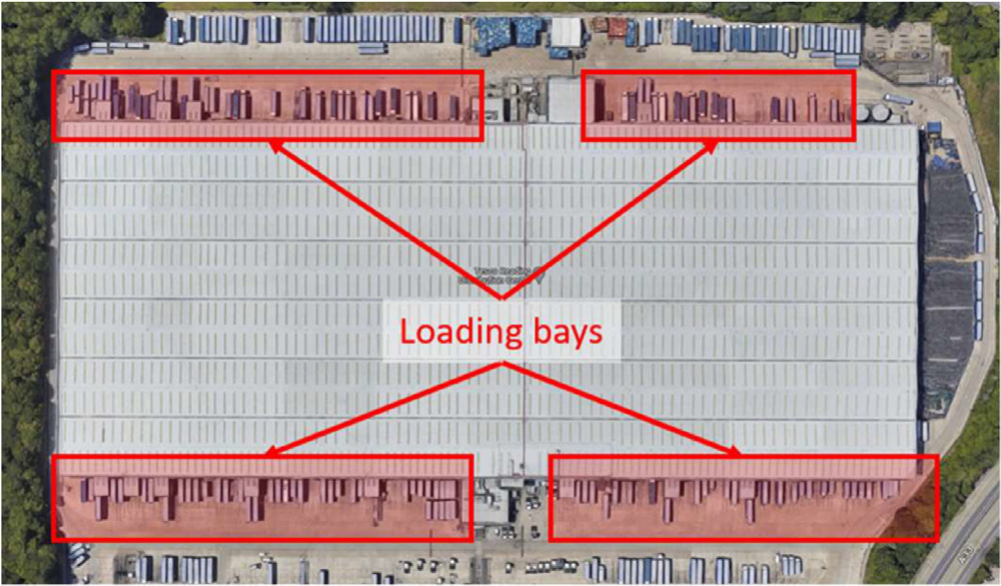
Satellite photo of the Tesco Distribution Centre in Reading, RG2 0PN, (warehouse site) with loading bays indicated.

**Fig. 4:**
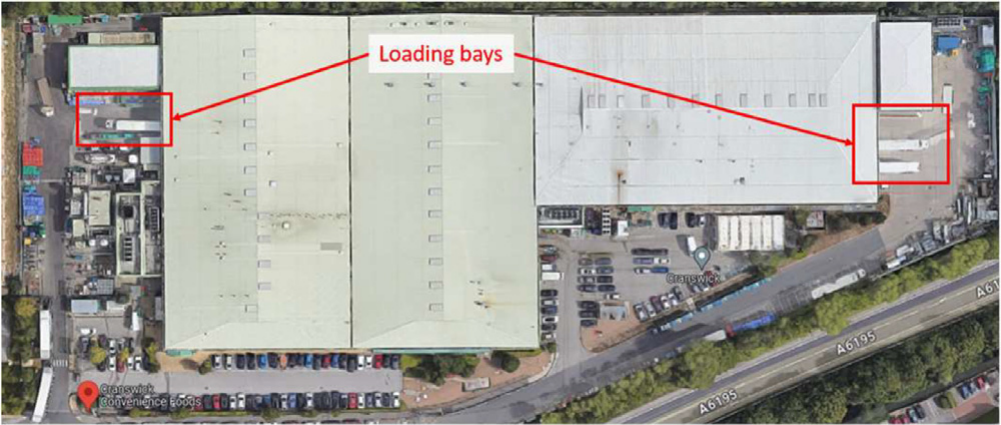
Satellite photo of Cranswick Convenience Foods, South Yorkshire, S73 0UN (factory site) with loading bays indicated.

**Fig. 5:**
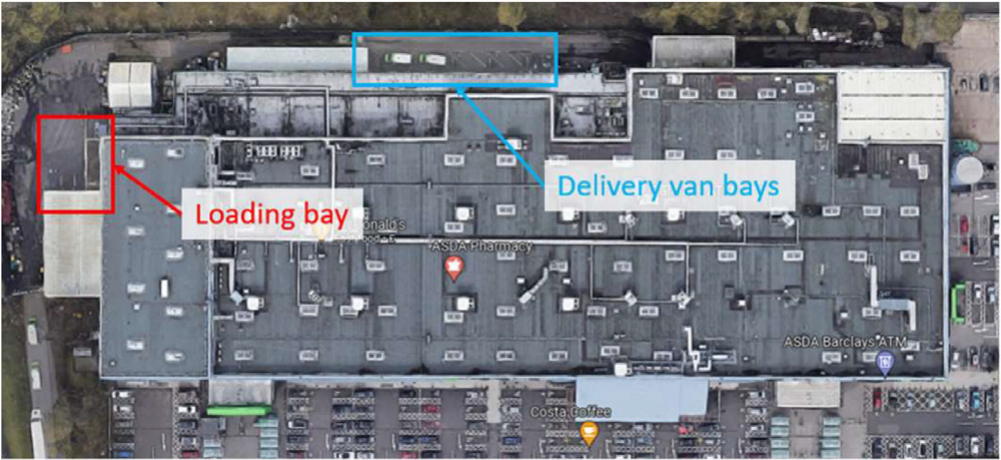
Satellite photo of ASDA, West Midlands, B76 8AD (retail site) with loading bays indicated.

Satellite images of one example site per category are shown in Figs. 3–5. In each case the loading bays are indicated. These were counted from the image manually. In addition to the standard aerial satellite view, the photogrammetric 3D view was used where available: to check the presence of loading bays in instances of uncertainty.

The first example, Fig. 3 shows one of the larger warehouse sites (an 80000 m^2^ Tesco distribution centre) with a very clear loading bay layout. The number of bays was found to be 174, and the VOA-reported and Google-measured floor areas agreed within 9%. This gives confidence that the site is a single-storey warehouse. The second example, Fig. 4, shows a Cranswick Convenience Foods factory site, with a VOA-reported size of 15,241 m^2^, a Google-measured size of 15,500 m^2^, and four visible loading bays. Finally, an example ASDA retail site in Fig. 5 is shown, with a VOA-reported size of 16,361 m^2^, a Google-measured footprint of 15,100 m^2^, and only one visible HGV loading bay.

Retail sites are expected to have one or two loading bays for inward deliveries to the store, and several more delivery van bays for outward deliveries to customers. This was the general observed trend, see Fig. 5. Note, however, that the focus of the work performed for this paper was HGV charging and consequently van bays were considered out of the current scope and were excluded from the loading bay count.

Also note in Fig. 3 the approximate occupancy rate of the bays. While such data were not collected in this study, further analysis on such a metric for these sites could help identify the potential number of battery chargers needed or which would be in use at any one time at a warehouse site. This could provide guidance on any necessary grid connection upgrades. Factories were the most difficult sites to identify loading bays, given the large variation in the size, shape and general layout, leading to irregular loading bay locations and markings.

#### 3.7.2 Results

**Fig. 6:**
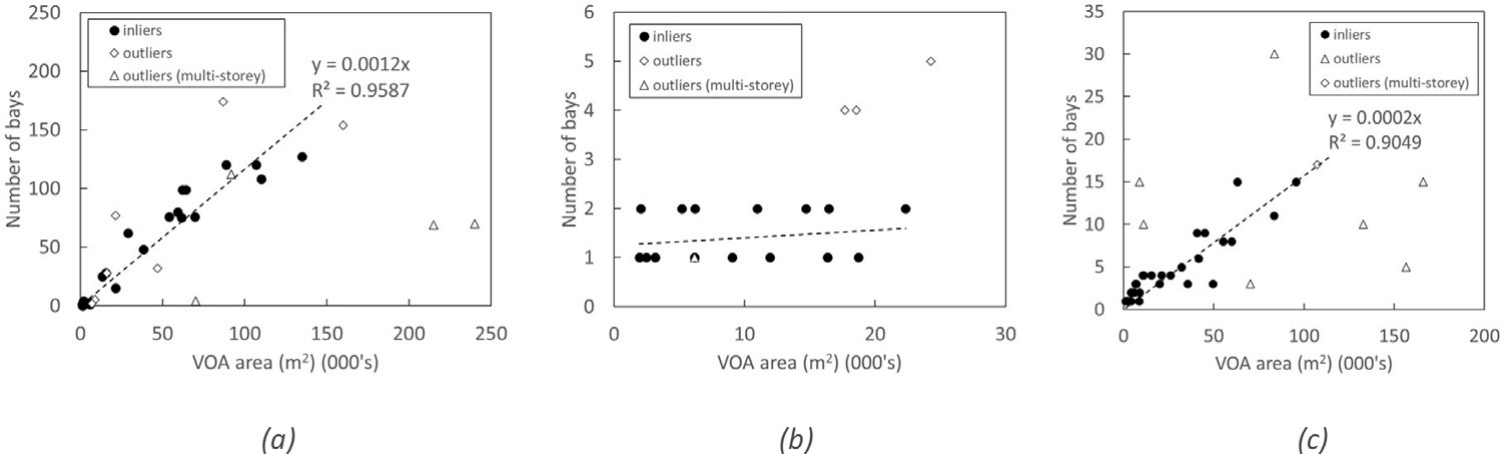
Loading bay to floor area correlations: (a) Warehouses, (b) Retail sites, (c) Factories.

Fig. 6 shows the results of the sampling and correlation exercise, with measured loading bays plotted against the VOA area in m^2^ for warehouses, retail sites and factories respectively. In each case the data are marked as either an inlier or outlier in accordance with the afore-mentioned requirements. The sample sizes were 36 for warehouses (24 inliers, 12 outliers), 22 for retail sites (19 inliers, 3 outliers), and 39 for factory sites (32 inliers, 7 outliers). In the case of the warehouse and factory sites, Fig. 6(a) and (c), a linear trend is evident. A zero-intercept has been forced in both cases, and only inliers were used for curve-fitting. In the case of retail sites, the trend is very different, with the vast majority of sites having only one or two bays. The three outliers are large home furnishing retail sites, namely an IKEA, a Costco, and a B&Q. In this case a assumption of either one or two loading bays per site could suffice for the model, though the trend favours one loading bay at lower areas where there is a higher umber of sites. For this reason we have assumed a conservative one bay per site, though users may choose to adopt an alternative model if needed.

Sites were known to be multistorey from the VOA record which indicates a breakdown of square meterage by floor where applicable. These accounted for 11% of sample warehouse data, 4% of sample retail data, and 5% of sample factory data. Other large discrepancies can be attributed to the difficulty in correctly discerning property boundaries on Google Maps (especially with complex factory sites). Smaller discrepancies can be attributed to the inherent accuracy of measuring the footprints from satellite data, leading to both small positive and negative errors between Google and VOA data, giving rise to the general spread of data around the trend lines.

Several of the deemed outliers in the data would in fact be reasonably well estimated from the trend lines (which were fitted only to the inlier data). I.e., only 6 out of 36 warehouse sites (16%) are significant outliers, 3 out of 22 for retail (13%) and 7 out of 39 for factories (18%). These give an indication of the potential accuracy of loading bay prediction for the rest of the dataset outside of the sample data. Factories are expected to give the highest errors overall due to the large variations in site layout and function.

**Fig. 7:**
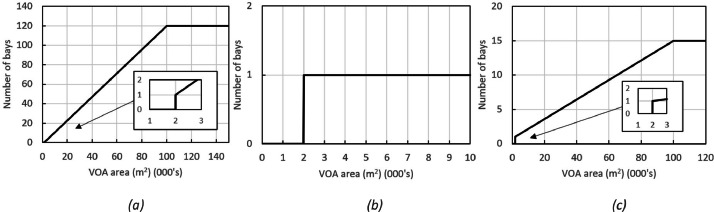
Loading bay to floor area models: (a) Warehouses, (b) Retail sites, (c) Factories.

The fitted models for the number of loading bays given the floor area are illustrated in Fig. 7(a) (b) (c).

For all three categories a lower threshold of 2000 m^2^ was adopted below which no HGV loading bay was assumed. This is based on empirical observations of sample sites from the dataset. A more detailed study of the processing capacity of logistics sites is recommended for further work to better understand which portion of sites are likely to be serviced by an HGV (and hence require a loading bay). Upper thresholds of 120 and 15 loading bays were enforced for warehouse and factory sites respectively. The upper threshold is particularly important for the largest warehouses sites where the VOA footprint could otherwise predict in excess of 300 loading bays, which is not realistic. For retail sites, one loading bay was assumed as discussed above.

Note that in the case of urban retail sites there is often no dedicated loading bay, even though these sites will likely be serviced daily by an HGV. In these cases, the HGV will often stop in the street outside the store during unloading. This is considered to count as a loading bay for the purposes of the current context, insofar as this is a proxy for a potential electric charging site.

## Limitations

• The dataset covers England and Wales and not Scotland or Northern Ireland, as limited by the raw data from the UK Valuation Office Agency.
• The VOA-reported floor area can be misleading for multistorey sites. The reported floor area is as per the valuation interpretation and so includes all floors of the structure, not just the land area (‘footprint’). As such these sites will likely present outliers in the loading bay correlation models. For a specific site, its listing on the valuation search portal will sometimes reveal whether it is multi-story or not in valuation calculation table.
• The loading bay data are based on models derived from a sample of the total dataset, and are not the only feasible models which could have been fitted. While they have been deemed suitably accurate for the current application, users may wish to derive alternative models as necessary for other applications and/or use larger samples of the data. Likewise the specification of a threshold footprint size is relevant to the current application of HGV-specific loading bay estimation. Other applications may require alternative thresholds to be determined though the same source data and methodology can be followed.
• The data may be improved for future work through increasing the sample sizes used to derive the loading bay models, using higher order models, or by considering more granular categorisation if necessary for the application, as necessary for the specific application.
• The source data do not contain any information on local electricity grid capacity to supply a given location. The data in this dataset would need to be coupled with additional data on grid connections and capacity for predicting the suitability of logistics sites for future HGV charging.

## Ethics Statement

The authors have read and follow the ethical requirements for publication in Data in Brief and confirm that the work does not involve human subjects, animal experiments, or any data collected from social media platforms.

## CRediT authorship contribution statement

**Christopher de Saxe:** Conceptualization, Methodology, Software, Formal analysis, Writing – original draft, Visualization. **Daniel Ainalis:** Methodology, Software, Writing – review & editing, Visualization, Project administration, Funding acquisition. **David Cebon:** Resources, Writing – review & editing, Supervision, Project administration, Funding acquisition.

## Data Availability

Logistics sites in England and Wales: Location, size, type and loading bays (Original data) (Apollo).
