# Supplementary material for: A dataset of logistics sites in England and Wales: Location, size, type and loading bays
Source: Data Brief. 2024 Apr 16;54:110399. doi: 10.1016/j.dib.2024.110399 (PMC11058711; doi:10.1016/j.dib.2024.110399)
Supplement: Supplementary file 1 [file mmc1.docx]

# Appendix A – Raw data description categories

The resultant descriptions per each of the three categories are presented in Table 2 (warehouses), Table 3 (factories), and Table 4 (retail). Entries are ordered according to their frequency of occurrence in the dataset. Both the number of occurrences in the category and relative frequency as a percentage of the total number of entries in the category are included.

Table 2: Descriptions categorised as ‘warehouses‘

| **Category** | **Freq.** | **Rel. freq.** | **Category** | **Freq.** | **Rel. freq.** | **Category** | **Freq.** | **Rel. freq.** |
| --- | --- | --- | --- | --- | --- | --- | --- | --- |
| warehouse and premises | 25648 | 92.62% | distribution warehouse and premises | 1 | 0.00% | warehouse used for retail and premises | 1 | 0.00% |
| warehouse, office and premises | 1096 | 3.96% | distribution depot & premises | 1 | 0.00% | warehouse yard and premises | 1 | 0.00% |
| cold store and premises | 157 | 0.57% | distribution warehouse & premises | 1 | 0.00% | warehouse, cold store & premises | 1 | 0.00% |
| warehouse, offices and premises | 155 | 0.56% | laboratories, warehouse and premises | 1 | 0.00% | warehouse, dock and premises | 1 | 0.00% |
| warehouse, workshop and premises | 131 | 0.47% | laboratories, warehouse, offices and premises | 1 | 0.00% | warehouse, factory and premises | 1 | 0.00% |
| warehouse & premises | 53 | 0.19% | laboratory warehouse and premises | 1 | 0.00% | warehouse, garage, office and premises | 1 | 0.00% |
| warehouses and premises | 52 | 0.19% | laboratory warehouse, office and premises | 1 | 0.00% | warehouse, laboratories and premises | 1 | 0.00% |
| warehouse offices and premises | 48 | 0.17% | laboratory, warehouse and premises | 1 | 0.00% | warehouse, laboratory and premises | 1 | 0.00% |
| warehouse | 32 | 0.12% | labs warehouse and premises | 1 | 0.00% | warehouse, meeting rooms and premises | 1 | 0.00% |
| warehouse, store and premises | 32 | 0.12% | railhead and distribution centre | 1 | 0.00% | warehouse, office and premises | 1 | 0.00% |
| distribution warehouse and premises | 29 | 0.10% | warehouse land and premises | 1 | 0.00% | warehouse, office & premises | 1 | 0.00% |
| warehouse showroom and premises | 21 | 0.08% | warehouse & premises | 1 | 0.00% | warehouse, office and premises (part exempt) | 1 | 0.00% |
| warehouse office and premises | 17 | 0.06% | warehouse & food production | 1 | 0.00% | warehouse, office storage land and premises | 1 | 0.00% |
| warehouse, showroom and premises | 14 | 0.05% | warehouse & land | 1 | 0.00% | warehouse, office, land and premises | 1 | 0.00% |
| cold stores and premises | 13 | 0.05% | warehouse & premises (pt derelict) | 1 | 0.00% | warehouse, offices and premises | 1 | 0.00% |
| cold store & premises | 8 | 0.03% | warehouse (part still reconstruction) | 1 | 0.00% | warehouse, offices & premises | 1 | 0.00% |
| coldstore and premises | 7 | 0.03% | warehouse (undergoing redevelopment) | 1 | 0.00% | warehouse, offices, land and premises | 1 | 0.00% |
| warehouse and premises. | 7 | 0.03% | warehouse / offices & premises | 1 | 0.00% | warehouse, retail and premises | 1 | 0.00% |
| warehouses, offices and premises | 7 | 0.03% | warehouse airfield and premises | 1 | 0.00% | warehouse, retail sales and premises | 1 | 0.00% |
| warehouse workshop and premises | 4 | 0.01% | warehouse and cafe | 1 | 0.00% | warehouse, shop and premises | 1 | 0.00% |
| distribution centre and premises | 3 | 0.01% | warehouse and cold stores and premises | 1 | 0.00% | warehouse, shop, cafe & premises | 1 | 0.00% |
| warehouse and premises | 3 | 0.01% | warehouse and factory and premises | 1 | 0.00% | warehouse, showroom & premises | 1 | 0.00% |
| warehouse and premises (part exempt) | 3 | 0.01% | warehouse and garden centre and premises | 1 | 0.00% | warehouse, showroom, office and premises | 1 | 0.00% |
| warehouse shop and premises | 3 | 0.01% | warehouse and premises [part exempt] | 1 | 0.00% | warehouse, trade centre and premises | 1 | 0.00% |
| warehouse, wharf and premises | 3 | 0.01% | warehouse and premises distribution | 1 | 0.00% | warehouse, training centre and premises | 1 | 0.00% |
| warehouse, workshops and premises | 3 | 0.01% | warehouse and premises(part exempt) | 1 | 0.00% | warehouse, workshop offices and premises | 1 | 0.00% |
| cold stores & premises | 2 | 0.01% | warehouse and premises, land and storage | 1 | 0.00% | warehouse, workshop, offices and premises | 1 | 0.00% |
| distribution depot and premises | 2 | 0.01% | warehouse and store and premises | 1 | 0.00% | warehouse, workshop, storage land and premises | 1 | 0.00% |
| warehouse office and premises | 2 | 0.01% | warehouse and training centre and premises | 1 | 0.00% | warehouse, workshop,offices and premises | 1 | 0.00% |
| warehouse offices and premises | 2 | 0.01% | warehouse brewery office and premises | 1 | 0.00% | warehouse, workshops ,offices and premises | 1 | 0.00% |
| warehouse ,offices and premises | 2 | 0.01% | warehouse cafe shop and premises | 1 | 0.00% | warehouse, workshops, offices & premises | 1 | 0.00% |
| warehouse and premise | 2 | 0.01% | warehouse cafes and premises | 1 | 0.00% | warehouse, workshops, offices and premises | 1 | 0.00% |
| warehouse factory and premises | 2 | 0.01% | warehouse function rooms and premises | 1 | 0.00% | warehouse,garage and premises | 1 | 0.00% |
| warehouse land and premises | 2 | 0.01% | warehouse glamping pods and premises | 1 | 0.00% | warehouse,office and premises | 1 | 0.00% |
| warehouse lorry park and premises | 2 | 0.01% | warehouse gymnasium and premises | 1 | 0.00% | warehouse,offices and premises | 1 | 0.00% |
| warehouse storage land and premises | 2 | 0.01% | warehouse hostel and premises | 1 | 0.00% | warehouse,offices,land & premises | 1 | 0.00% |
| warehouse wharf and premises | 2 | 0.01% | warehouse office & premises | 1 | 0.00% | warehouse,shop and premises | 1 | 0.00% |
| warehouse, cold store and premises | 2 | 0.01% | warehouse offices & premises | 1 | 0.00% | warehouse,showroom and premises | 1 | 0.00% |
| warehouse, land and premises | 2 | 0.01% | warehouse petrol filling station and premises | 1 | 0.00% | warehouse,showroom & premises | 1 | 0.00% |
| warehouse, showroom and premises | 2 | 0.01% | warehouse production area and premises | 1 | 0.00% | warehouse,showroom and premises | 1 | 0.00% |
| warehouse, storage land and premises | 2 | 0.01% | warehouse sales area and premises | 1 | 0.00% | warehouse,storage yard and premises | 1 | 0.00% |
| warehouse, supermarket, restaurant and and premises | 2 | 0.01% | warehouse scrap yard and premises | 1 | 0.00% | warehouse,workshop and premises | 1 | 0.00% |
| chill store, warehouse & premises | 1 | 0.00% | warehouse showroom & premises | 1 | 0.00% | warehouses | 1 | 0.00% |
| cold store warehouses & premises | 1 | 0.00% | warehouse showroom offices and premises | 1 | 0.00% | warehouses & premises | 1 | 0.00% |
| cold store, factory and premises | 1 | 0.00% | warehouse store and premises | 1 | 0.00% | warehouses chill stores & premises | 1 | 0.00% |
| cold store, warehouse & premises | 1 | 0.00% | warehouse stores and premises | 1 | 0.00% | warehouses offices and premises | 1 | 0.00% |
| cold store, warehouse and premises | 1 | 0.00% | warehouse trade counter and premises | 1 | 0.00% | warehouses, office and premises | 1 | 0.00% |
| cold stores, factory and premises | 1 | 0.00% | warehouse trade shop and premises | 1 | 0.00% | warehouses, offices and premises (part exempt) | 1 | 0.00% |
| coldstore and remises | 1 | 0.00% | warehouse training facility office and premises | 1 | 0.00% | warehouses, stores and premises | 1 | 0.00% |
| coldstore, packhouse hostel & premises (pt exempt) | 1 | 0.00% | warehouse used as gymnasium & premises | 1 | 0.00% | warehouses, workshops and premises | 1 | 0.00% |
| distillery warehouse & premises | 1 | 0.00% | warehouse used as roller skating rink and premises | 1 | 0.00% |  |  |  |

Table 3: Descriptions categorised as ‘factories’

| **Category** | **Freq.** | **Rel. freq.** | **Category** | **Freq.** | **Rel. freq.** | **Category** | **Freq.** | **Rel. freq.** |
| --- | --- | --- | --- | --- | --- | --- | --- | --- |
| retail warehouse and premises | 3847 | 59.73% | retail warehouse, workshop and premises | 3 | 0.05% | showroom warehouse and premises | 1 | 0.02% |
| superstore and premises | 2043 | 31.72% | wholesale warehouse | 2 | 0.03% | showroom, offices, warehouse & premises | 1 | 0.02% |
| supermarket and premises | 229 | 3.56% | wholesale warehouse & premises | 2 | 0.03% | showroom, warehouse & premises | 1 | 0.02% |
| wholesale warehouse and premises | 67 | 1.04% | aquatic superstore and premises | 1 | 0.02% | showroom, warehouse, stores and premises | 1 | 0.02% |
| car supermarket and premises | 60 | 0.93% | builders merchant and warehouse | 1 | 0.02% | showroom, warehouse, workshop and premises | 1 | 0.02% |
| superstore petrol filling station and premises | 56 | 0.87% | car delivery warehouse office and premises | 1 | 0.02% | supermarket & premsies | 1 | 0.02% |
| superstore, petrol filling station and premises | 19 | 0.29% | car supermarkets & premises | 1 | 0.02% | supermarket and pemises | 1 | 0.02% |
| retail warehouse & premises | 17 | 0.26% | cash & carry, warehouse, office and premises | 1 | 0.02% | supermarket, petrol filling station and premises | 1 | 0.02% |
| showroom, warehouse and premises | 17 | 0.26% | retail warehouse | 1 | 0.02% | superstore and premises | 1 | 0.02% |
| retail warehouse, store and premises | 13 | 0.20% | retail warehouse and premises | 1 | 0.02% | superstore petrol filling station and premises | 1 | 0.02% |
| supermarket & premises | 10 | 0.16% | retail warehouse and premises. | 1 | 0.02% | superstore petrol station and premises | 1 | 0.02% |
| superstore, store and premises | 6 | 0.09% | retail warehouse garden centre and premises | 1 | 0.02% | superstore, petrol filling station and premises | 1 | 0.02% |
| retail warehouse and premises | 5 | 0.08% | retail warehouse, car space and premises | 1 | 0.02% | superstore, petrol filling station & premises | 1 | 0.02% |
| retail warehouse, office and premises | 4 | 0.06% | retail warehouse, offices and premises | 1 | 0.02% | superstore, petrol station and premises | 1 | 0.02% |
| showroom warehouse and premises | 4 | 0.06% | retail, warehouse and premises | 1 | 0.02% | trade warehouse showroom and premises | 1 | 0.02% |
| superstore & premises | 4 | 0.06% | shop warehouse and premises | 1 | 0.02% |  |  |  |
| trade warehouse and premises | 4 | 0.06% | shop, warehouse and premises | 1 | 0.02% |  |  |  |

Table 4: Descriptions categorised as ‘retail’

| **Category** | **Freq.** | **Rel. freq.** | **Category** | **Freq.** | **Rel. freq.** | **Category** | **Freq.** | **Rel. freq.** |
| --- | --- | --- | --- | --- | --- | --- | --- | --- |
| factory and premises | 13073 | 96.47% | factory and premises | 1 | 0.01% | factory, retail outlet and premises | 1 | 0.01% |
| factory, office and premises | 256 | 1.89% | factory and premises (in course of redevelopment) | 1 | 0.01% | factory, shop and premises | 1 | 0.01% |
| factory, workshop and premises | 74 | 0.55% | factory and premises (part exempt) | 1 | 0.01% | factory, showrooms and premises | 1 | 0.01% |
| factory, offices and premises | 41 | 0.30% | factory and premises (part fire damaged) | 1 | 0.01% | factory, stores and premises | 1 | 0.01% |
| factory & premises | 18 | 0.13% | factory and premises in builders hands | 1 | 0.01% | factory, warehouse, and premises | 1 | 0.01% |
| factory, store and premises | 12 | 0.09% | factory and premises partly under redevleopment | 1 | 0.01% | factory, wharf and premises | 1 | 0.01% |
| factory, warehouse and premises | 6 | 0.04% | factory and premises. | 1 | 0.01% | factory, workshop, warehouse and premises | 1 | 0.01% |
| factory office and premises | 5 | 0.04% | factory and premisesderelict | 1 | 0.01% | factory, workshops, storage land and premises | 1 | 0.01% |
| factory and premise | 4 | 0.03% | factory cold store and premises | 1 | 0.01% | factory,reservoir and premises | 1 | 0.01% |
| factory offices and premises | 3 | 0.02% | factory laboratory and premises | 1 | 0.01% | factory,warehouse,offices & premises | 1 | 0.01% |
| factories and premises | 2 | 0.01% | factory offices & premises | 1 | 0.01% | factory,workshop & premises | 1 | 0.01% |
| factories, workshop and warehouse | 2 | 0.01% | factory showroom and premises | 1 | 0.01% | factory/offices and premises | 1 | 0.01% |
| factory/warehouse and premises | 2 | 0.01% | factory used as 5 a side football centre | 1 | 0.01% | factory/warehouse | 1 | 0.01% |
| bakery warehouse and premises | 1 | 0.01% | factory warehouse and premises | 1 | 0.01% | food processing factory and premises | 1 | 0.01% |
| brewery warehouse and premises | 1 | 0.01% | factory workshops and premises | 1 | 0.01% | football factory and premises | 1 | 0.01% |
| brewery warehouse, office and premises | 1 | 0.01% | factory, farm shop and premises | 1 | 0.01% | fruit packing depot and premises (part exempt) | 1 | 0.01% |
| brewery, warehouse, stores and premises | 1 | 0.01% | factory, food hall, cafe and premises | 1 | 0.01% | offices, factory and premises | 1 | 0.01% |
| dairy, warehouse and premises | 1 | 0.01% | factory, garden centre and premises | 1 | 0.01% | packhouse, warehouse and premises | 1 | 0.01% |
| factory | 1 | 0.01% | factory, museum, shop and premises | 1 | 0.01% | paper mill factory and premises | 1 | 0.01% |
| factory and premises | 1 | 0.01% | factory, office, warehouse and premises | 1 | 0.01% | smokehouse factory | 1 | 0.01% |
| factory & premises | 1 | 0.01% | factory, offices & premises | 1 | 0.01% | vacant factory and premises | 1 | 0.01% |
| factory ,and premises | 1 | 0.01% | factory, pub and premises | 1 | 0.01% | wafer fabrication factory & premises | 1 | 0.01% |
